# Gemcitabine induces Parkin-independent mitophagy through mitochondrial-resident E3 ligase MUL1-mediated stabilization of PINK1

**DOI:** 10.1038/s41598-020-58315-w

**Published:** 2020-01-30

**Authors:** Ryoko Igarashi, Shun-ichi Yamashita, Tomohiro Yamashita, Keiichi Inoue, Tomoyuki Fukuda, Takeo Fukuchi, Tomotake Kanki

**Affiliations:** 1 Department of Cellular Physiology, Niigata University Graduate School of Medical and Dental Sciences, Niigata, 951-8510 Japan; 2 Department of Global Healthcare, Graduate School of Pharmaceutical Sciences, Kyushu University, Fukuoka, Japan; 3 Department of Ophthalmology, Niigata University Graduate School of Medical and Dental Sciences, Niigata, 951-8510 Japan

**Keywords:** Mitophagy, Mitochondria

## Abstract

Mitophagy plays an important role in the maintenance of mitochondrial homeostasis. PTEN-induced kinase (PINK1), a key regulator of mitophagy, is degraded constitutively under steady-state conditions. During mitophagy, it becomes stabilized in the outer mitochondrial membrane, particularly under mitochondrial stress conditions, such as in treatment with uncouplers, generation of excessive mitochondrial reactive oxygen species, and formation of protein aggregates in mitochondria. Stabilized PINK1 recruits and activates E3 ligases, such as Parkin and mitochondrial ubiquitin ligase (MUL1), to ubiquitinate mitochondrial proteins and induce ubiquitin-mediated mitophagy. Here, we found that the anticancer drug gemcitabine induces the stabilization of PINK1 and subsequent mitophagy, even in the absence of Parkin. We also found that gemcitabine-induced stabilization of PINK1 was not accompanied by mitochondrial depolarization. Interestingly, the stabilization of PINK1 was mediated by MUL1. These results suggest that gemcitabine induces mitophagy through MUL1-mediated stabilization of PINK1 on the mitochondrial membrane independently of mitochondrial depolarization.

## Introduction

Autophagy is a bulk degradation process of cytosolic components and is highly conserved from yeast to mammals. Upon induction of autophagy, a membrane sac termed the isolation membrane, emerges at its nucleation site. Isolation membrane formation requires autophagy-related (ATG) proteins, including the ULK1 complex, the Class III phosphatidylinositol (PI) 3-kinase complex, and the PI 3-phosphate binding WIPI proteins. The isolation membrane elongates and engulfs cytosolic components, and then its edge is closed, which gives rise to the double-membrane structure, termed the autophagosome. Finally, the autophagosome fuses with the lysosome, forming the autolysosome, where lysosomal hydrolases degrade the internal components^1,2^.

In addition to bulk degradation, certain cellular components can be recognized as cargos for selective autophagy, including protein aggregates, invading bacteria, and damaged organelles^3–5^. Mitochondria are also degraded by selective autophagy, which is called mitophagy^6^. Upon induction of mitophagy, ATG proteins recognize the portion of mitochondria destined for degradation through ubiquitin-dependent or -independent mechanisms^7,8^.

In ubiquitin-dependent mitophagy, outer mitochondrial membrane (OMM) proteins of the damaged mitochondria are initially ubiquitinated, followed by the recruitment of autophagy-adaptor proteins, such as optineurin and NDP52, to the mitochondria via binding to the ubiquitinated OMM proteins. Such adaptor proteins also bind to ATG proteins and recruit them to the mitochondria, ensuring the formation of the isolation membrane^9^. The mechanism by which OMM proteins are ubiquitinated under mitochondrial stress conditions has been well studied by examining serine/threonine PTEN-induced kinase (PINK1) and Parkin-mediated mitophagy^10–12^. Under steady-state conditions, PINK1 is transported into the mitochondria and laterally released in the inner mitochondrial membrane (IMM) through its mitochondrial targeting sequence (MTS) and transmembrane domain. In the IMM, the N-terminus of PINK1 is cleaved by presenilins-associated rhomboid-like protein (PARL), an IMM-resident protease, and then cleaved PINK1 is retro-translocated to the cytoplasm and eventually degraded by the proteasome. Since the membrane potential (∆Ψm) across the IMM is the driving force required for importing MTS-containing PINK1 into the IMM, upon ∆Ψm loss, PINK1 cannot be transported to the IMM and instead is stabilized in the OMM through its OMM localization signal^13,14^. Stabilized PINK1 induces the translocation of Parkin, an E3 ubiquitin ligase, to the mitochondria, leading to ubiquitination of OMM proteins^10,11,15^. As well as the mitochondrial depolarization that is induced by mitochondrial uncouplers, such as carbonyl cyanide m-chlorophenyl hydrazone (CCCP), it has been reported that generation of excessive reactive oxygen species and accumulation of misfolded mitochondrial proteins in the mitochondria can cause the stabilization of PINK1 and induce a Parkin-mediated mitophagy^16,17^. Notably, under mitochondrial misfolded protein stress conditions, stabilization of PINK1 is not accompanied by mitochondrial depolarization, suggesting that PINK1 is stabilized not only by mitochondrial depolarization but also potentially by other unidentified mechanisms^17^. Since stabilization of PINK1 is a critical step for ensuring the specificity of damaged mitochondria for mitophagy, it is important to understand the mechanisms underlying stabilization of PINK1 following different types of mitochondrial stress.

In this study, we find that gemcitabine, an anticancer drug, induces the stabilization of PINK1 and subsequent mitophagy in HeLa cells in which Parkin is not expressed. Following gemcitabine treatment, mitochondrial E3 ubiquitin protein ligase 1 **(**MUL1) is required for the stabilization of PINK1 and is upstream of PINK1. Here, we propose a novel mechanism by which PINK1 is stabilized in the OMM in a MUL1-dependent manner after gemcitabine treatment.

## Results

### Gemcitabine induces mitophagy in HeLa cells in a PINK1-dependent manner

**Figure 1:**
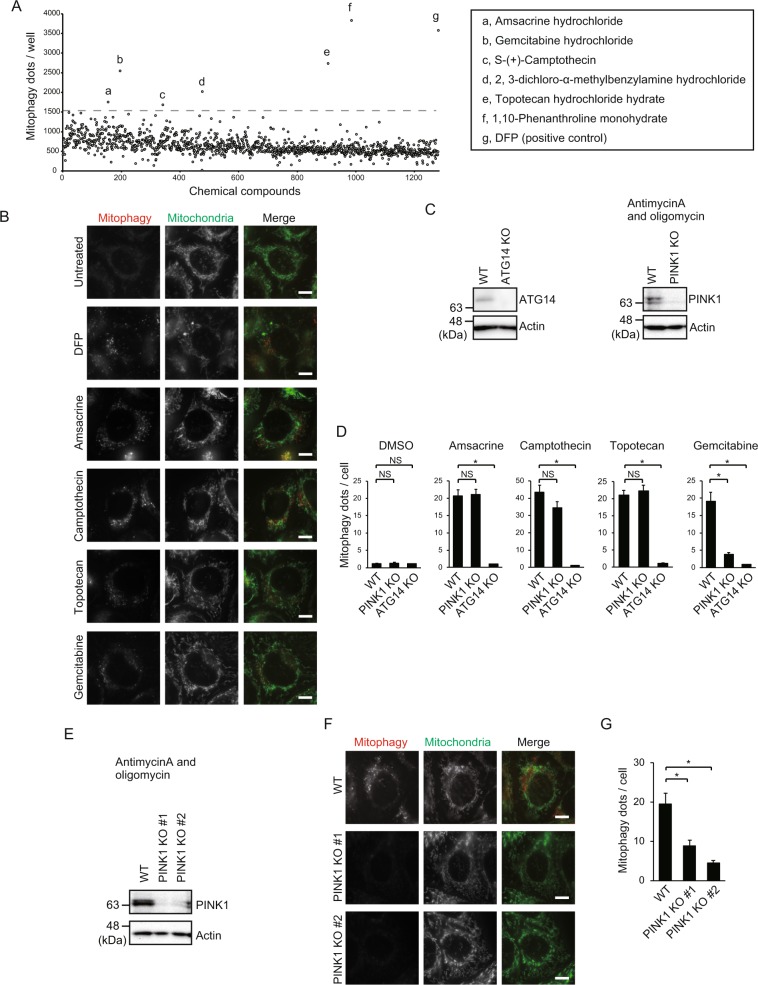
Gemcitabine induces mitophagy in HeLa cells in a PINK1 dependent manner. (**A**) Screening of the LOPAC1280 library for mitophagy-inducing chemicals. Chemicals that potently induce mitophagy are listed. (**B**) Fluorescence in HeLa cells expressing mt-Keima measured by two excitation filters: mt-Keima is excited by 590-nm light in lysosomes, indicating mitophagy (red), and excited by 440-nm light in mitochondrial matrix, indicating mitochondria (green). Bars, 10 µm. (**C**) Immunoblot showing the loss of PINK1 and ATG14 proteins in the KO cell lines. Full-length blots are presented in Supplementary Fig. S1A–D. (**D**) Mitophagy dots were calculated from at least 47 cells treated with indicated chemicals. Data are shown as the mean ± SEM. The data is a representative of three independent experiments. Similar results were obtained in the other two experiments. *p < 0.05; NS, not significant. (**E**) Immunoblot showing the loss of PINK1 in two PINK1 KO cell lines. WT and PINK1 KO cell lines were cultured in the presence of 4 µM antimycinA and 10 µM oligomycin for 3 h to stabilize PINK1 prior to immunoblot. Full-length blots are presented in Supplementary Fig. S1A, B. (**F**) Loss of mitophagy in two PINK1 KO cell lines after gemcitabine treatment, analyzed by fluorescence microscopy as in B. Bars, 10 µm. (**G**) Mitophagy dots shown in F were calculated from at least 50 cells treated with gemcitabine. Data are shown as the mean ± SEM. The data is a representative of three independent experiments. Similar results were observed in the other two experiments. *p < 0.05.

Mitophagy is important for controlling cellular functions in response to environmental stimuli by eliminating excessive or damaged mitochondria^18^, but the molecular mechanisms are not fully understood. To identify novel mitophagic stimuli, we established a high-throughput screening system using HeLa cells stably expressing the mitochondria-targeting Keima protein (mt-Keima). As previously described, the mitophagy assay, using the mt-Keima system, enables the highly sensitive detection of mitophagy signals without fixation and staining^19–21^. Using this system, we screened the LOPAC1280 chemical library, containing over 1000 chemicals and identified six mitophagy-inducing agents (Fig. 1A). Since Parkin is not expressed in HeLa cells^22^, these chemicals induce mitophagy through a Parkin-independent pathway. We focused on four anticancer chemicals out of six because 1,10-Phenanthroline is an already-known mitophagy inducer and 2,3-dichloro-α-methylbenzylamine hydrochloride was unreproducible in a subsequent analysis (data not shown)^23^. Treatment with the anticancer drugs at 10 μM induced mitophagy similarly to 1 mM deferiprone (DFP), a well-known mitophagy-inducing agent (Fig. 1B). Next, we further investigated the ability of these chemicals to induce mitophagy in ATG14 or PINK1 knockout (KO) cell lines (Fig. 1C, Supplementary Fig. S1A–D). As expected, mitophagy was completely inhibited in ATG14 KO cells regardless of the chemical used, indicating that these chemicals induce mitophagy through conventional autophagy machinery (Fig. 1D). Intriguingly, upon gemcitabine treatment, mitophagy was significantly decreased in PINK1 KO cells, unlike the other drugs (Fig. 1D). Two other PINK1 KO cell lines were generated by CRISPR/Cas9 system to rule out off-target effects. The loss of PINK1 protein was confirmed by immunoblotting (Fig. 1E, Supplementary Fig. S1A,B). Upon gemcitabine treatment, mitophagy signals were significantly decreased in both PINK1 KO cell lines, confirming that PINK1 is required for gemcitabine-induced mitophagy (Fig. 1F,G). Furthermore, to investigate whether the mitochondria is specifically enwrapped by autophagosomes upon gemcitabine treatment, we carried out immunofluorescence analysis with anti-Tom20 and anti-LC3 as markers for the mitochondria and autophagosomes, respectively. In this context, to clearly detect autophagosomes, cells were cultured with bafilomycin A1 which inhibits fusion between autophagosomes and lysosomes and leads to the accumulation of autophagosomes in the cytoplasm. Tom20 puncta were observed and colocalized with LC3 after gemcitabine treatment (Supplementary Fig. S1E, upper panels). Colocalization was not detected in untreated cells and PINK1 KO cells (Supplementary Fig. S1E, lower panels). As mentioned above, Parkin is dispensable for gemcitabine-induced mitophagy, which prompted us to investigate whether mitochondrial proteins are ubiquitinated upon gemcitabine treatment. To address this concern, we stained gemcitabine treated cells with ubiquitin antibody. Unlike CCCP treatment of Parkin-expressing HeLa cells, no obvious ubiquitin signals on the mitochondria were detected even after gemcitabine treatment (Supplementary Fig. S1G,F). These data suggest that gemcitabine induces PINK1-dependent mitophagy, but independently of mitochondrial amplified ubiquitination.

### PINK1 is stabilized by gemcitabine independently of mitochondrial depolarization

**Figure 2:**
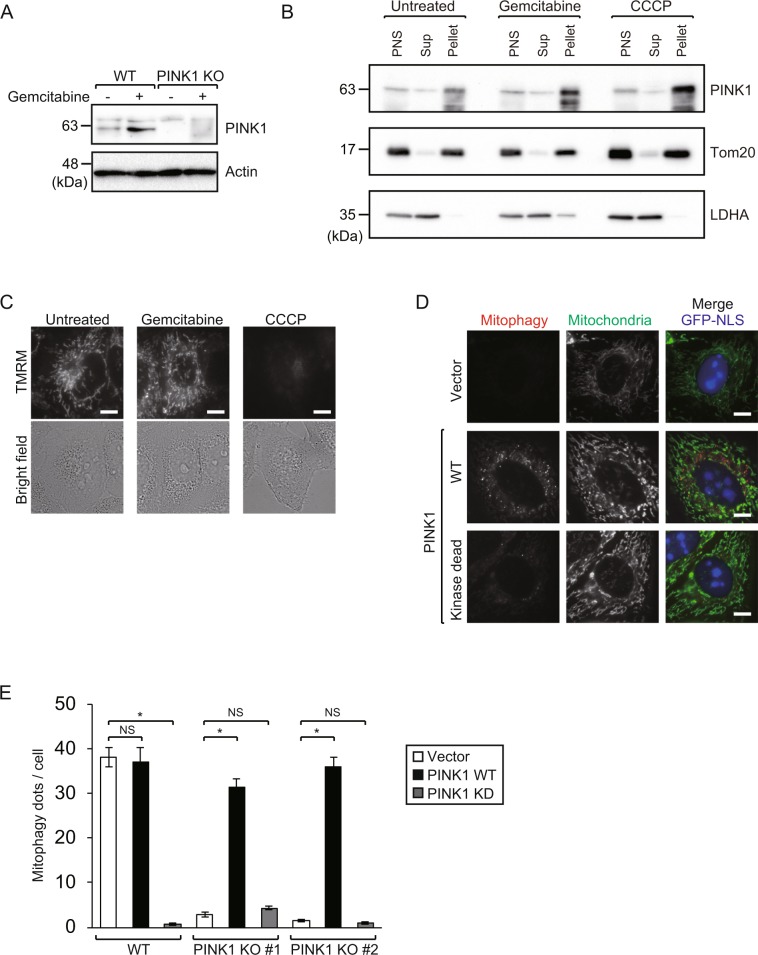
PINK1 is stabilized by gemcitabine independently of mitochondrial depolarization. (**A**) PINK1 immunoblot for the WT and PINK1 KO cell line # 1 following culture with or without gemcitabine for 48 h. Full-length blots are presented in Supplementary Fig. S2A, B. (**B**) HeLa cells were treated with 100 μM gemcitabine for 48 h or 10 μM CCCP for 3 h and then subjected to subcellular fractionation. All fractions were processed by immunoblotting with anti-PINK1, anti-Tom20 (mitochondria marker), and anti-LDHA (cytoplasm marker) antibodies. PNS, Sup, and pellet represent the post-nuclear supernatant, 10000 × g supernatant fraction, and 10000 × g pellet fraction, respectively. Full-length blots are presented in Supplementary Fig. S2C–E. (**C**) HeLa cells were cultured with or without 100 μM gemcitabine for 24 h or treated with CCCP. Cells were stained with TMRM and analyzed by fluorescence microscopy. Bars, 10 µm. (**D**) WT and two PINK1 KO cell lines were transfected with IRES-GFP-NLS empty vector, PINK1 WT or PINK1 KD vectors and cultured with 100 μM gemcitabine for 48 h. Mitophagy was analyzed by fluorescence microscopy as in Fig. 1B. Fluorescence images of PINK1 KO # 1 are shown. Bars, 10 µm. (**E**) Mitophagy dots shown in D were calculated from at least 20 transfected cells with nuclear GFP signal. Data are shown as the mean ± SEM. The data is a representative of three independent experiments. Similar results were observed in the other two experiments. *p < 0.05; NS, not significant.

In conventional ubiquitin-dependent mitophagy, PINK1 is stabilized on the depolarized mitochondria and then mediates recruitment and activation of downstream E3 ligases to ensure ubiquitination of OMM proteins^14^. We first investigated whether gemcitabine treatment induces PINK1 stabilization. We performed immunoblot analysis with an anti-PINK1 antibody using total cell lysates from gemcitabine-treated or untreated HeLa cells. Consistent with previous reports, a faint detection band for PINK1 was observed in untreated cells due to the constitutive degradation of PINK1 under steady-state conditions. Intriguingly, after gemcitabine treatment, the detection intensity of stabilized PINK1 increased, compared to the untreated control. (Fig. 2A, Supplementary Fig. S2A,B). To further investigate where PINK1 is stabilized by gemcitabine treatment, we carried out subcellular fractionation experiments for PINK1. Similar to CCCP treatment, stabilized PINK1 was primarily detected in the mitochondria enriched fraction after gemcitabine treatment (Fig. 2B, Supplementary Fig. S2C–E). These results suggest that gemcitabine stabilizes PINK1 associated with mitochondria. To address the question of whether stabilization of PINK1 is due to mitochondrial depolarization, we examined the mitochondrial membrane potential (∆Ψm) using Tetramethylrhodamine Methyl Ester (TMRM), a ∆Ψm-dependent fluorescent dye^11,15^. After gemcitabine treatment, TMRM staining of the mitochondria was similar to untreated cells, suggesting that, unlike CCCP, stabilization of PINK1 by gemcitabine is not accompanied by mitochondrial depolarization (Fig. 2C).

### PINK1 kinase activity is required for gemcitabine-induced mitophagy

Since PINK1 kinase activity is required for the translocation of Parkin to the mitochondria during ubiquitin-mediated mitophagy^10,24^, we investigated whether PINK1 kinase activity is required for gemcitabine-induced mitophagy. We cloned PINK1 cDNA into the IRES-GFP-NLS expression plasmid and introduced kinase-dead (KD) mutation (K219A, D362A, and D364A) to abolish its kinase activity^25^. PINK1 KO cells were transfected with an empty vector, wild-type PINK1 (PINK1 WT), or PINK1 KD, and subsequently treated with gemcitabine. PINK1 WT, but not PINK1 KD, rescued mitophagy levels to a similar extent as WT cells (Fig. 2D,E), suggesting that PINK1 kinase activity is required for gemcitabine-induced mitophagy. Exogenous PINK1 KD expression also disrupted mitophagy in WT cells, suggesting that PINK1 KD has a dominant-negative effect on gemcitabine-induced mitophagy (Fig. 2E).

### MUL1 is required for gemcitabine-induced mitophagy

**Figure 3:**
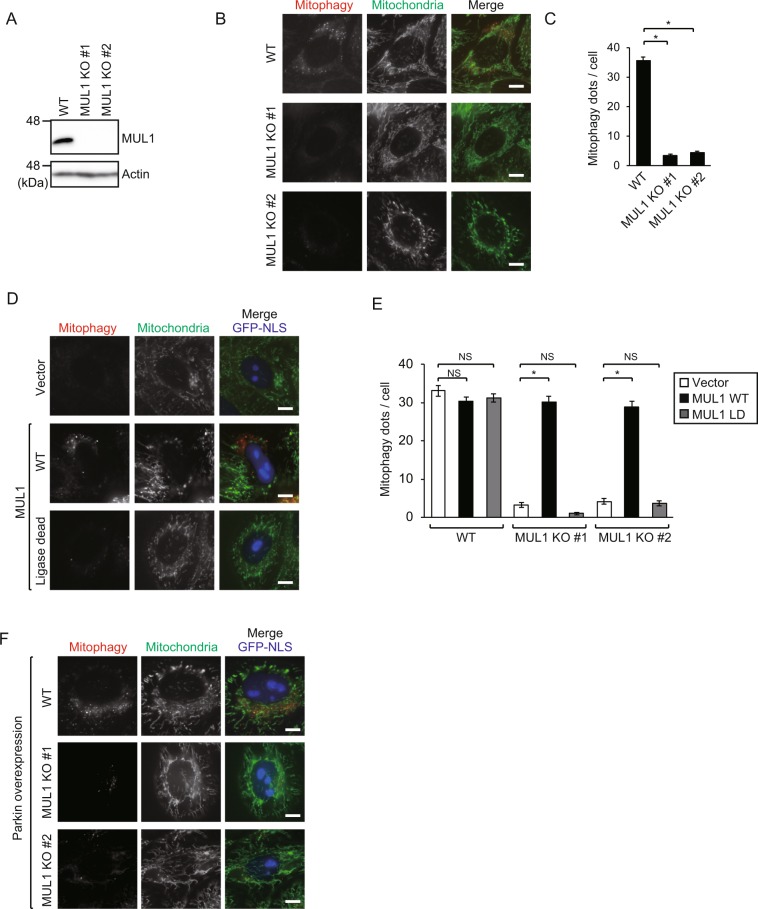
MUL1 is required for gemcitabine-induced mitophagy. (**A**) MUL1 immunoblot showing the loss of MUL1 in two different MUL1 KO cell lines. Full-length blots are presented in Supplementary Fig. S3A, B. (**B**) WT and two MUL1 KO HeLa cells were cultured with gemcitabine for 48 h. Mitophagy was analyzed by fluorescence microscopy as in Fig. 1B. Bars, 10 µm. (**C**) Mitophagy dots shown in B were calculated from at least 40 cells treated with gemcitabine. Data are shown as the mean ± SEM. The data is a representative of three independent experiments. Similar results were observed in the other two experiments. *p < 0.05; NS, not significant. (**D**) WT and two MUL1 KO cell lines were transfected with IRES-GFP-NLS empty vector, MUL1 WT or MUL1 LD vectors and cultured with 100 μM gemcitabine for 48 h. Mitophagy was analyzed by fluorescence microscopy as in Fig. 1B. Fluorescence images of MUL1 KO # 1 are shown. Bars, 10 µm. (**E**) Mitophagy dots shown in D were calculated from at least 20 transfected cells with nuclear GFP signal. Data are shown as the mean ± SEM. The data is a representative of three independent experiments. Similar results were observed in the other two experiments. *p < 0.05; NS, not significant. (**F**) WT and two MUL1 KO cell lines were transfected with Parkin-IRES-GFP-NLS vector and cultured with gemcitabine for 48 h. Mitophagy was analyzed by fluorescence microscopy as in Fig. 1B. Bars, 10 µm.

Since HeLa cells do not express Parkin, another ubiquitin ligase might likely be involved in the gemcitabine-induced mitophagy pathway. MUL1 has been previously reported to have a role in mitophagy^26,27^. To evaluate the role of MUL1 on mitophagy, we generated two MUL1 KO cell lines using CRISPR-Cas9 and verified the loss of MUL1 protein by immunoblotting (Fig. 3A, Supplementary Fig. S3A,B). Upon gemcitabine treatment, mitophagy signals were significantly decreased in both MUL1 KO cell lines, suggesting that MUL1 is required for gemcitabine-induced mitophagy (Fig. 3B,C).

Next, we investigated whether MUL1 E3 ligase activity is required. We cloned MUL1 cDNA into the IRES-GFP-NLS vector and introduced a ligase-dead (LD) mutation (H319A) to abolish E3 ligase activity^27^. MUL1 KO cell lines were transfected with wild-type MUL1 (MUL1 WT) or ligase-dead MUL1 (MUL1 LD) and subsequently treated with gemcitabine to evaluate whether mitophagy could be rescued by these constructs. Mitophagy was not rescued by MUL1 LD in MUL1 KO cells, unlike MUL1 WT, which rescued mitophagy to a similar level as in WT cells (Fig. 3D,E), suggesting that MUL1 E3 ligase activity is required for gemcitabine-induced mitophagy. MUL1 was reported to function redundantly with Parkin in mitophagy^26^. However, upon gemcitabine treatment, overexpression of Parkin was not able to rescue mitophagy in MUL1 KO cells (Fig. 3F), suggesting that the function of MUL1 in gemcitabine-induced mitophagy is different from previously reported^26,27^.

### MUL1 is involved in the stabilization of PINK1

**Figure 4:**
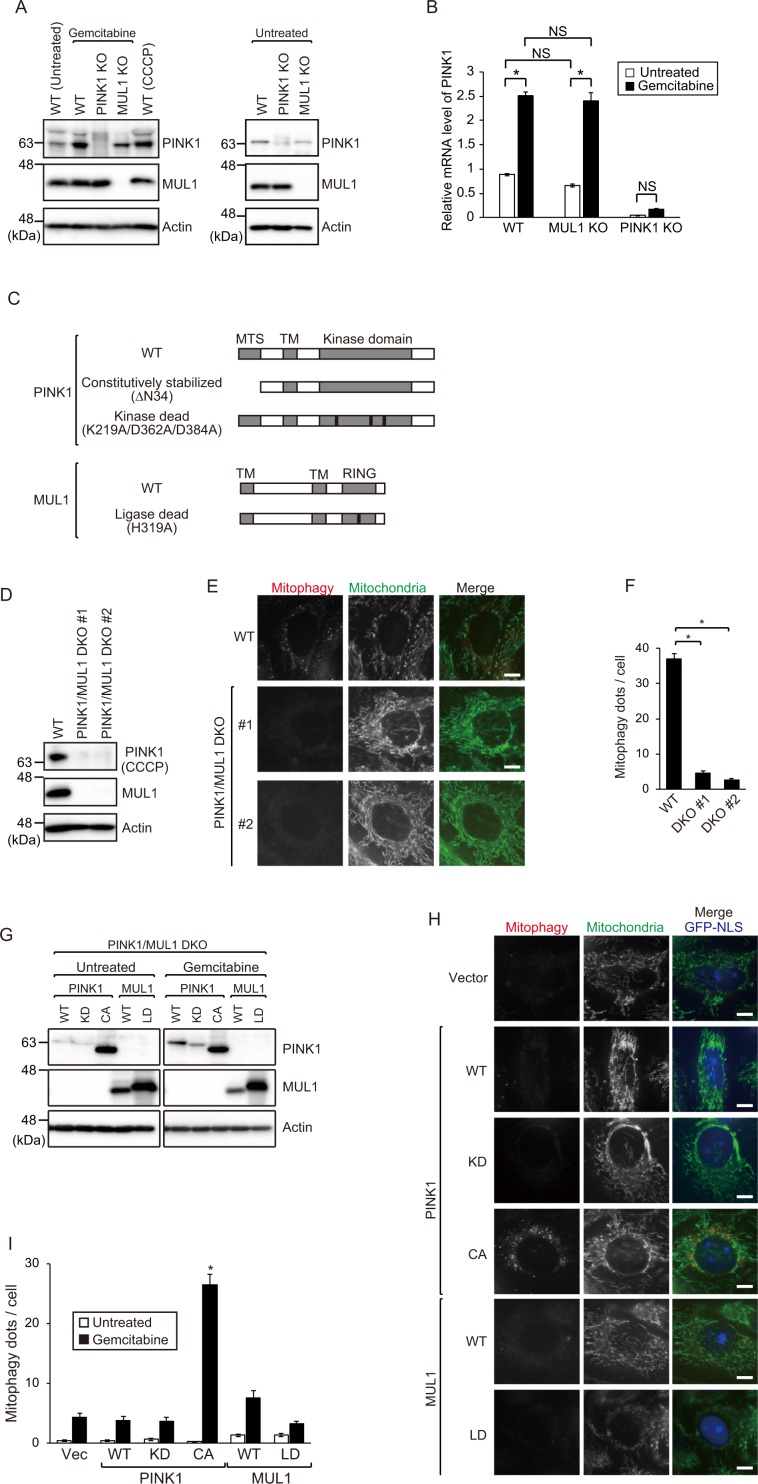
MUL1 is involved in the stabilization of PINK1. (**A**) WT, PINK1 KO # 1, and MUL1 KO # 1 cell lines were cultured with or without gemcitabine for 48 h. Cells were lysed and analyzed by immunoblotting with anti-PINK1, anti-MUL1, and anti-Actin antibodies. Full-length blots are presented in Supplementary Fig. S4A–C. (**B**) PINK1 mRNA expression levels in WT, MUL1 KO, and PINK1 KO cells after gemcitabine treatment. Data are shown as the mean ± SEM. *p < 0.05; NS, not significant, determined with one-way ANOVA followed by the Tukey-Kramer *post hoc* test. (**C**) Schematic diagrams of PINK1 and MUL1 variants used. PINK1 kinase-dead mutant harbors K219A/D362A/D384A mutations in its kinase domain. MUL1 ligase-dead mutant harbors an H319A mutation in RING finger domain. (**D**) Immunoblot for PINK1, MUL1, and actin showing loss of PINK1 and MUL1 in two different PINK1/MUL1 DKO cell lines. WT and two PINK1/MUL1 DKO HeLa cell lines were cultured in the presence of 10 µM CCCP for 3 h to stabilize PINK1. Full-length blots are presented in Supplementary Fig. S4D–F. (**E**) WT and two PINK1/MUL1 DKO HeLa cells were cultured with gemcitabine for 48 h. Mitophagy was analyzed by fluorescence microscopy as in Fig. 1B. Bars, 10 µm. (**F**) Mitophagy dots shown in E were calculated from at least 40 cells treated with gemcitabine. Data are shown as the mean ± SEM. The data is a representative of three independent experiments. Similar results were observed in the other two experiments. *p < 0.05. (**G, H**) PINK1/MUL1 DKO # 1 HeLa cells were transfected with IRES-GFP-NLS empty vector, PINK1 and MUL1 variants listed in C and cultured with gemcitabine for 48 h. Cells were subjected to immunoblotting with anti-PINK1, anti-MUL1, anti-GFP, and anti-Actin antibodies (**G**), and their mitophagy were analyzed by fluorescence microscopy as in Fig. 1B. Bars, 10 µm (**H**). Full-length blots are presented in Supplementary Fig. S4G–J. (**I**) Mitophagy dots shown in H were calculated from at least 20 transfected cells with nuclear GFP signal. Data are shown as the mean ± SEM. The data is a representative of three independent experiments. Similar results were observed in the other two experiments. *p < 0.05.

To examine the relationship between PINK1 and MUL1, we first investigated the stabilization of PINK1 by gemcitabine treatment in MUL1 KO cells. Following gemcitabine treatment, the level of stabilized PINK1 was lower in MUL1 KO cells than in WT cells, suggesting that MUL1 is an upstream factor involved in the stabilization of PINK1 (Fig. 4A, Supplementary Fig. S4A–F). To address how MUL1 stimulates PINK1 stabilization upon gemcitabine treatment, we investigated the direct effect of gemcitabine and the contribution of MUL1 on the mRNA expression levels of PINK1. Interestingly, PINK1 mRNA levels were increased in gemcitabine treated cells, but this transcriptional upregulation was detected in MUL1 KO cells to a same extent as WT cells. This strongly suggests that MUL1 facilitates PINK1 stabilization at the post-transcriptional level (Fig. 4B). To confirm these observations, we expressed several mutants of PINK1 and MUL1 in PINK1/MUL1 DKO HeLa cells (Fig. 4C). Since the N-terminal 34 amino acid deletion mutant of PINK1 (MTS-deleted PINK1, PINK1∆N34) has been reported to stabilize it in the OMM even under steady-state conditions and induce Parkin-dependent mitophagy due to loss of PINK1 import into the IMM, we used PINK1∆N34 as a constitutively stabilized form of PINK1^13^. We first generated PINK1/MUL1 DKO HeLa cells and verified the loss of each protein by immunoblotting (Fig. 4D, Supplementary Fig. S4G–I). Gemcitabine-induced mitophagy was significantly decreased in PINK1/MUL1 DKO cell lines as compared to WT (Fig. 4E,F). PINK1/MUL1 DKO cells were then transfected with five different variants of PINK1 or MUL1 constructs, as shown in Fig. 4C, and cultured in the presence or absence of gemcitabine. The protein expression level and mitophagic activity of the transfected cells were analyzed by immunoblotting and mt-Keima assay, respectively. PINK1∆N34 was stabilized even in the absence of MUL1, whereas the stabilized PINK1s were decreased in the context of the other PINK1 variants even following gemcitabine treatment (Fig. 4G, Supplementary Fig. S4J–M), further confirming that MUL1 is required for PINK1 stabilization. Upon gemcitabine treatment, PINK1∆N34, but not other variants, rescued mitophagy in PINK1/MUL1 DKO cells (Fig. 4H,I). This suggests that MUL1 acts upstream of PINK1 by facilitating PINK1 stabilization. Notably, unlike in Parkin-mediated mitophagy where expression of PINK1∆N34 is sufficient to induce mitophagy^13^, although PINK1∆N34 is stabilized in both untreated and gemcitabine treated cells, PINK1∆N34 induced mitophagy only after gemcitabine treatment (Fig. 4G–I, Supplementary Fig. S4J–M). Taken together, these results suggest that, upon gemcitabine treatment, MUL1 stabilizes PINK1 and then unknown downstream factor(s) of PINK1 is activated by gemcitabine in a MUL1-independent manner.

## Discussion

**Figure 5:**
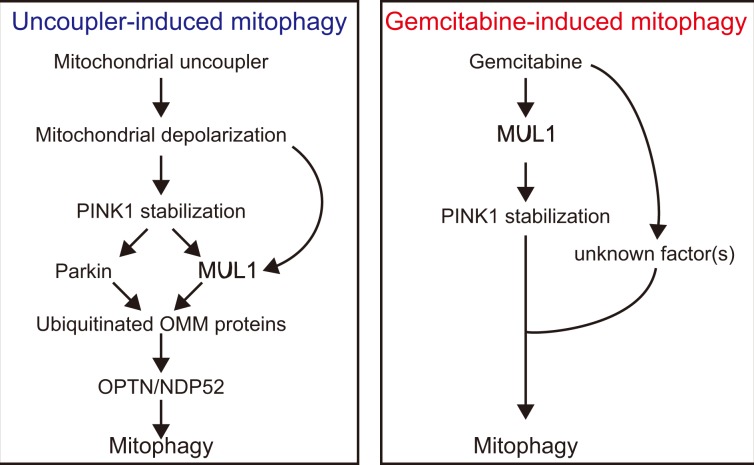
Representative schemes for mitochondrial uncoupler-induced mitophagy and gemcitabine-induced mitophagy.

In the present study, we screened a chemical library for mitophagy inducers and discovered that gemcitabine induces PINK1 stabilization and subsequent mitophagy even in the absence of Parkin. Notably, the stabilization of PINK1 was not accompanied by mitochondrial depolarization. We also found that MUL1 plays an important role in PINK1 stabilization in the OMM. Hence, we conclude that gemcitabine treatment induces a novel type of PINK1-dependent Parkin-independent mitophagy, where MUL1 facilitates PINK1 stabilization (Fig. 5).

Currently, two other studies have reported a relation among MUL1, PINK1, and Parkin. One showed that MUL1 acts in parallel to the PINK1-Parkin axis in *Drosophila*^27^, and the other showed that MUL1 and Parkin function redundantly as downstream factors of PINK1 in the mouse embryo^26^. In the present study, we propose an alternative relationship among them, in which MUL1 acts upstream of PINK1 in a Parkin-independent pathway.

Since gemcitabine does not cause mitochondrial depolarization, gemcitabine-induced PINK1 stabilization is likely to take place differently from that of mitochondrial ∆Ψm loss-induced mitophagy. PINK1∆N34, which fails to be imported into the IMM due to the loss of MTS^13^, overcomes the limitation of stabilization of PINK1 and mitophagy in MUL1 KO cells, suggesting that MUL1 prevents PINK1 import into the IMM. MUL1 has an ubiquitin and small ubiquitin-like modifier (SUMO) E3 ligase activity toward Mfn and Drp1, respectively, positively regulating mitochondrial fission through these protein modifications^27–32^. Because the E3 ligase activity of MUL1 is required for gemcitabine-induced mitophagy, mitochondrial fission might be involved in the prevention of PINK1 import into the IMM. Since MUL1 can SUMOylate other mitochondrial substrates as well as Drp1^28^, it is also possible that unknown SUMOylated mitochondrial proteins may contribute to PINK1 stabilization. Moreover, MUL1 maintains endoplasmic reticulum (ER)-mitochondria contact through the downregulation of mitofusin 2^30^. Thus, we speculate that ER-mitochondria contact may mediate PINK1 stabilization.

Under steady-state conditions, PINK1 is imported into the IMM through the translocase of the OMM (TOM) complex and the translocase of the IMM (TIM) complex^14^. In the IMM, PINK1 is cleaved by PARL and subsequently retro-translocated to the cytoplasm. To elucidate which step of the PINK1 metabolism is modulated by MUL1, further investigation regarding the import activity of the TIM and TOM complexes and the PARL protease activity toward PINK1 upon gemcitabine treatment will be needed.

Following mitochondrial depolarization, stabilized PINK1 is activated through the formation of a complex with the TOM complex and subsequent auto-phosphorylation^33,34^. Although PINK1 KD is stabilized after mitochondrial depolarization, it does not form a high-molecular weight complex. Because PINK1 KD overexpression inhibited gemcitabine-induced mitophagy in WT cells, the assembly of the complex containing endogenous PINK1 is likely to be impaired by exogenous PINK1 KD during gemcitabine treatment. Although PINK1∆N34, a constitutively active form of PINK1, restored gemcitabine-induced mitophagy in the absence of MUL1, PINK1∆N34 per se did not induce mitophagy in untreated cells. This is in contrast to the Parkin-mediated pathway, where PINK1∆N34 induces Parkin recruitment and mitophagy without any stimuli. Therefore, unknown downstream factor(s) of PINK1 that are activated by gemcitabine may be involved in gemcitabine-induced mitophagy. ARIH and SIAH, two other E3 ligases that are downstream of PINK1^35,36^, may be activated by gemcitabine treatment and promote mitophagy.

## Materials and Methods

### Mitophagy assay

Keima is a pH-sensitive fluorescent protein that, in a neutral environment (i.e., mitochondrial matrix), is excited by a 440-nm light (shown in green), but not by a 590-nm light. When the Keima protein is present in acidic conditions, (i.e., autolysosomes), it is excited by a 590-nm light (shown in red), but not by a 440-nm light. HeLa cells or mutant cells that stably expressed mt-Keima were cultured at 37 °C under 5% CO_2_ (Yamashita *et al*. 2016. J Cell Biol). After mitophagy induction, cells were analyzed by fluorescence microscopy with two excitation filters (430 ± 24-nm and 560 ± 40), and detection was performed at a 624 ± 20-nm emission.

### Screening of the chemical library

Cells expressing mt-Keima were seeded in 100 µl of media containing 1,000 cells per well in 96-well optical bottom plates (165305, Thermo Scientific) using the Multidrop Combi reagent dispenser (5840300, Thermo Scientific) and cultured at 37 °C for 24 h. Chemical compounds were dissolved in DMSO and added to the cultures at 10 µM using the Biomek NXP automated liquid handler (A16094, Beckman Coulter Life Sciences). DMSO or 1 mM DFP was added as negative and positive controls, respectively. The plates were incubated for 24 h at 37 °C under 5% CO_2_ and analyzed using the IN Cell Analyzer 2000 (GE Healthcare Life Sciences). The fluorescence images of mt-Keima signals in the lysosome, which are specifically excited at 590 nm, were analyzed using the IN Cell Developer Toolbox.

### Antibodies and reagents

The following primary antibodies were used: anti-PINK1 rabbit polyclonal (BC100-494, Novus), anti-PINK1 rabbit monoclonal (6946 S, Cell Signaling Technology), anti-MUL1 rabbit monoclonal (ab209263, abcam), anti-LC3 mouse monoclonal (CTB-LC3-2-IC, Cosmo Bio), anti-ATG14 mouse monoclonal (M184-3, Medical and Biological Laboratories), anti-Multi Ubiquitin mouse monoclonal (D058-3, Medical and Biological Laboratories), anti-Tom20 mouse monoclonal (sc-17764, Santa Cruz Biotechnology), anti-LDHA mouse monoclonal (sc-137243, Santa Cruz Biotechnology), and anti-Actin mouse monoclonal (MAB1501R, Millipore). The following reagents were used: DFP (3-Hydroxy-1,2-dimethyl-4(1 H)-pyridone) (322–65152), oligomycin (O4533), antimycin A (#514-55521), CCCP (carbonyl cyanide 3-chlorophenylhydrazone) (034–16993), and hygromycin B (089–06151), Tetramethylrhodamine Methyl Ester (TMRM) (203–18041) were purchased from FUJIFILM Wako Pure Chemical. The Library of Pharmacologically Active Compounds (LOPAC1280) (LO4200), amsacrine (A9809), camptothecin (C9911), gemcitabine (G6423), and topotecan (T2705) were purchased from Sigma–Aldrich. Bafilomycin A1 (BVT-0252) was purchased from Adipogen Life Sciences.

### Cell culture, induction of mitophagy, and transfection

HeLa cells were cultured in Dulbecco’s Modified Eagle’s Medium (043–30085, FUJIFILM Wako Pure Chemical) supplemented with 10% fetal bovine serum (10270, Gibco) at 37 °C under 5% CO_2_. To induce mitophagy by chemical treatment, HeLa cells stably expressing mt-Keima were cultured in media supplemented with 1 mM DFP, 10 µM amsacrine, 10 µM camptothecin, 10 µM topotecan, or 10 µM gemcitabine for 24 h (shown in Fig. 1). To distinguish mutant phenotypes clearly, cells were cultured with 100 μM gemcitabine for 48 h (shown in Figs. 2–4). To stabilize PINK1 by loss of ∆Ψm, HeLa cells were cultured in media supplemented with 10 µM CCCP or 4 µM antimycin A and 10 µM oligomycin for 3 h. DNA transfection was performed using FuGENE HD (Promega), according to the manufacturer’s instructions.

### Generation of KO HeLa cell lines using the CRISPR/Cas9 system

To generate the KO HeLa cell lines, the following guide RNA sequences were used: PINK1 #1 (5′-GCCGGGCCTACGGCTTGGGG-3′), PINK1 #2 (5′-GTTGGACACGAGACGCTTGC-3′), MUL1 #1 (5′-GCCGCCCTGTACTCCGTGTAC-3′), MUL1 #2 (5′-GTACTCCGTGTACCGGCAGA-3′). CRISPR plasmids were constructed with pX330-U6-Chimeric_BB-CBh-hSpCas9 (42230, Addgene), as previously described (Yamashita *et al*. 2016. J Cell Biol). HeLa cells were co-transfected with CRISPR vector and pcDNA3.1-Hyg (−) (V87520, Invitrogen). Twenty-four hours after transfection, the media was replaced with hygromycin B-containing media and cultured for 3 days to select for positively transfected cells. Next, cells were cloned by limiting dilution in 96 well plates in normal media conditions. Clonal populations were screened by immunoblotting.

### Immunoblotting

Cells were cultured in 24 well plates and then lysed with 1 × SDS sample buffer to obtain total cell lysates. Samples were sonicated and then heated for 5 min at 95 °C. Lysates were separated by SDS-PAGE and were then transferred to a PVDF membrane (IPVH00010, Millipore) by a semi-dry blotting system. Blots were blocked with 5% skimmed milk in PBS-Tween20 (PBS-T) for 30 min at room temperature and then incubated with primary antibodies diluted in 2% skimmed milk in PBS-Tween20 overnight at 4 °C. After incubation with HRP-conjugated secondary antibodies, blots were incubated with HRP substrate (2332638, ATTO), and images were analysed by ChemiDoc (Bio-rad Laboratories).

### Subcellular fractionation

Cells were suspended with homogenization buffer (20 mM HEPES pH 7.4, 250 mM Sucrose, 1 mM EDTA, 1 mM PMSF and protease inhibitor cocktail) and lysed by passing the cells through a 25-gauge needle 10 times. Post nuclear supernatant (PNS) was prepared by a centrifugation at 800 × g for 5 min. To prepare the mitochondria enriched pellet fraction, PNS was further centrifuged at 10000 × g for 20 min.

### Construction of plasmids and mutagenesis

cDNA encoding *PINK1* and *MUL1* were amplified by PCR from HeLa cells cDNA library and cloned into the BamHI-NotI sites of the IRES-GFP-NLS vector (Otera *et al*. 2010. J Cell Biol). To introduce the kinase dead mutations (K219A/D362A/D384A) in PINK1 and the ligase dead mutation (H319A) in MUL1, the following primers were used: PINK1 K219AFw, 5′-CCC TTG GCC ATC GCG ATG ATG TGG AAC ATC-3′; PINK1 K219Arv, 5′-GAT GTT CCA CAT CAT CGC GAT GGC CAA GGG-3′; PINK1 D362AFw, 5′-CAT CGC GCA CAG AGC CCT GAA ATC CGA C-3′; PINK1 D362ARv, 5′-GTC GGA TTT CAG GGC TCT GTG CGC GAT G-3′; PINK1 D384AFw, 5′-GCT GGT GAT CGC AGC TTT TGG CTG CTG C-3′; PINK1 D384ARv, 5′-GCA GCA GCC AAA AGC TGC GAT CAC CAG C-3′; MUL1 H319AFw, 5′-CTG GAG TGT GGG GCC GTT TGT TCC TGC ACC-3′; MUL1 H319ARv, 5′-GGT GCA GGA ACA AAC GGC CCC ACA CTC CAG-3′. Mutations were introduced by inverse PCR-based mutagenesis.

### RNA preparation and quantitative PCR

Cells were cultured in the presence and absence of gemcitabine. Total RNA was then extracted with TRIzol reagent (Thermo Fisher Scientific), according to the manufacturer’s instructions. cDNAs were synthesized from total RNA using ReverTraAce qPCR RT Kit (TOYOBO Life Science); then, real-time PCR reactions were performed using the THUNDERBIRD SYBR qPCR Mix (TOYOBO Life Science) and a Thermal Cycler Dice Real Time system II (TAKARA) with following primers; PINK1 Fw, 5′-TCCAAGAGAGGTCCCAAGCA-3′; PINK1 Rv, 5′-AGGGCAGCACATCAGGGTAG-3′; Actin Fw, 5′-AGAGCTACGAGCTGCCTGAC-3′; Actin Rv, 5′-AGCACTGTGTTGGCGTACAG-3′).

### Statistical analysis

All quantifications were performed from three biologically independent experimental replicates, with similar data variances observed among groups. Unless otherwise noted, statistical analyses were performed using the Kruskal-Wallis test followed by the Steel and the Steel-Dwass *post hoc* tests to assess significance. P < 0.05 was considered statistically significant and was indicated by the use of one asterisk.

## Supplementary information

Supplementary Information.

Supplementary Information 2.
