# Supplementary material for: Gemcitabine induces Parkin-independent mitophagy through mitochondrial-resident E3 ligase MUL1-mediated stabilization of PINK1
Source: Sci Rep. 2020 Jan 30;10:1465. doi: 10.1038/s41598-020-58315-w (PMC6992789; doi:10.1038/s41598-020-58315-w)
Supplement: Supplementary file 1 — Supplementary Information. [file 41598_2020_58315_MOESM1_ESM.pdf]

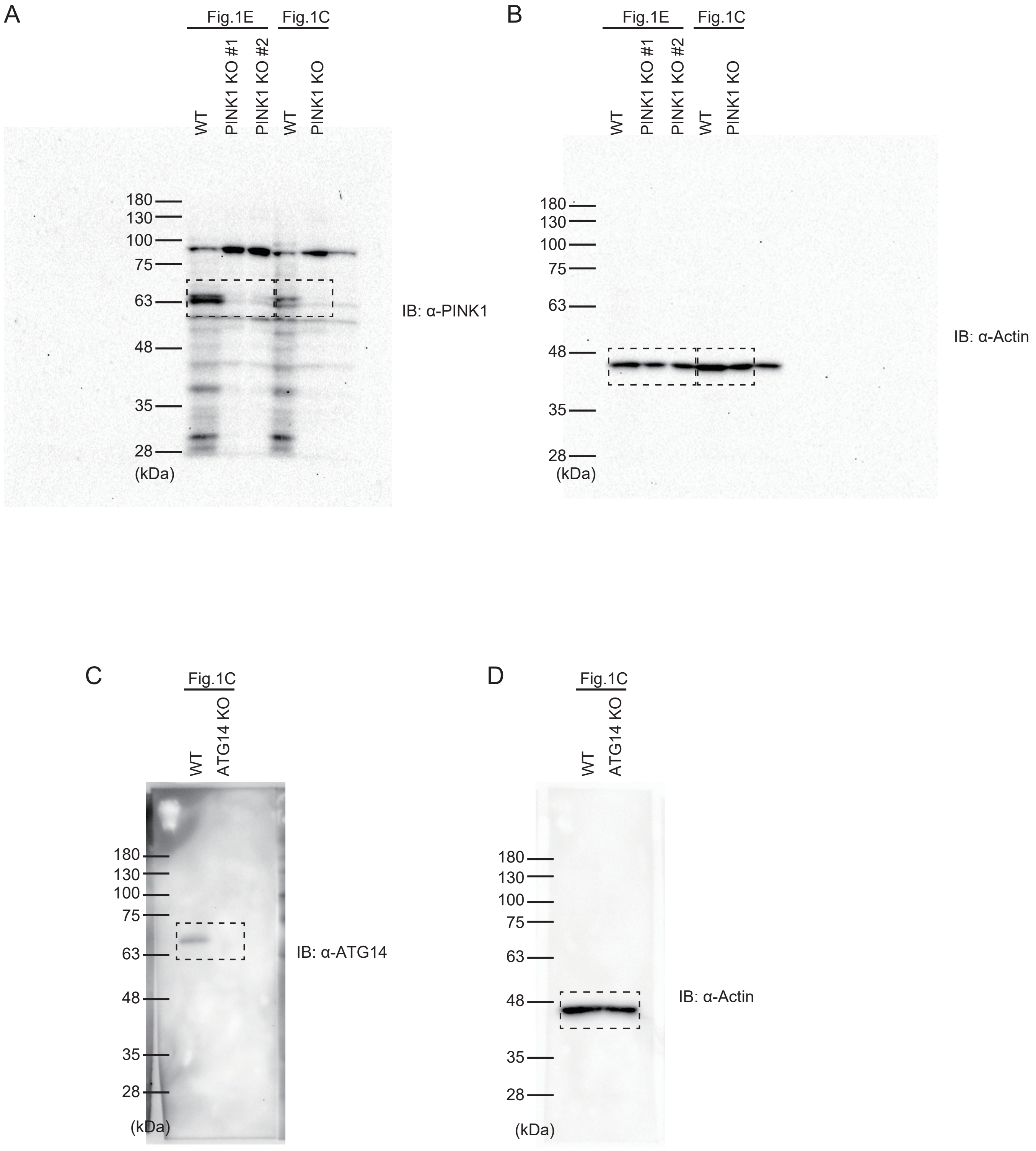
A
Fig.1E
Fig.1C
WT
PINK1 KO #1
PINK1 KO #2
WT
PINK1 KO
180
130
100
75
63
48
35
28
(kDa)
IB: α-PINK1
B
Fig.1E
Fig.1C
WT
PINK1 KO #1
PINK1 KO #2
WT
PINK1 KO
180
130
100
75
63
48
35
28
(kDa)
IB: α-Actin
C
Fig.1C
WT
ATG14 KO
180
130
100
75
63
48
35
28
(kDa)
IB: α-ATG14
D
Fig.1C
WT
ATG14 KO
180
130
100
75
63
48
35
28
(kDa)
IB: α-Actin

Figure S1

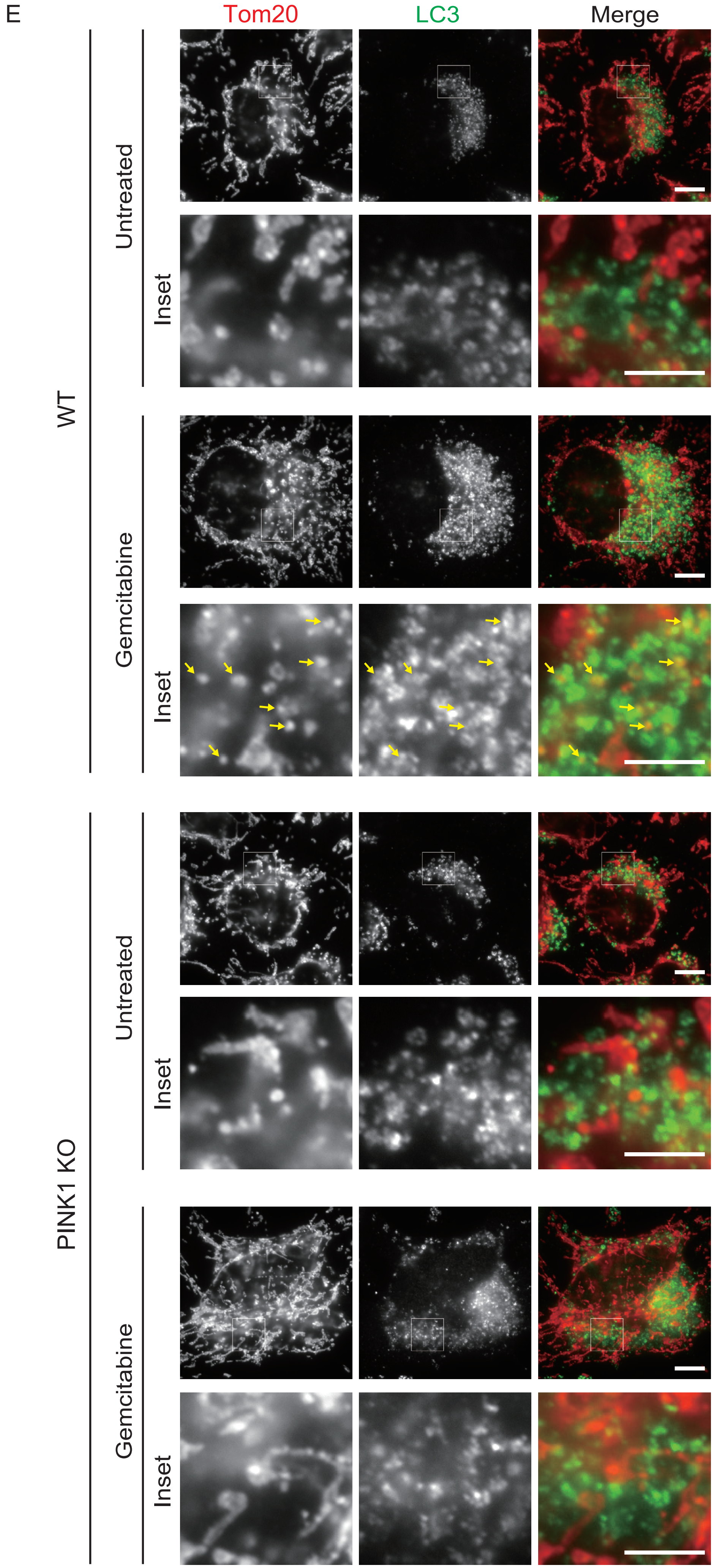

Figure S1 (continued)

F

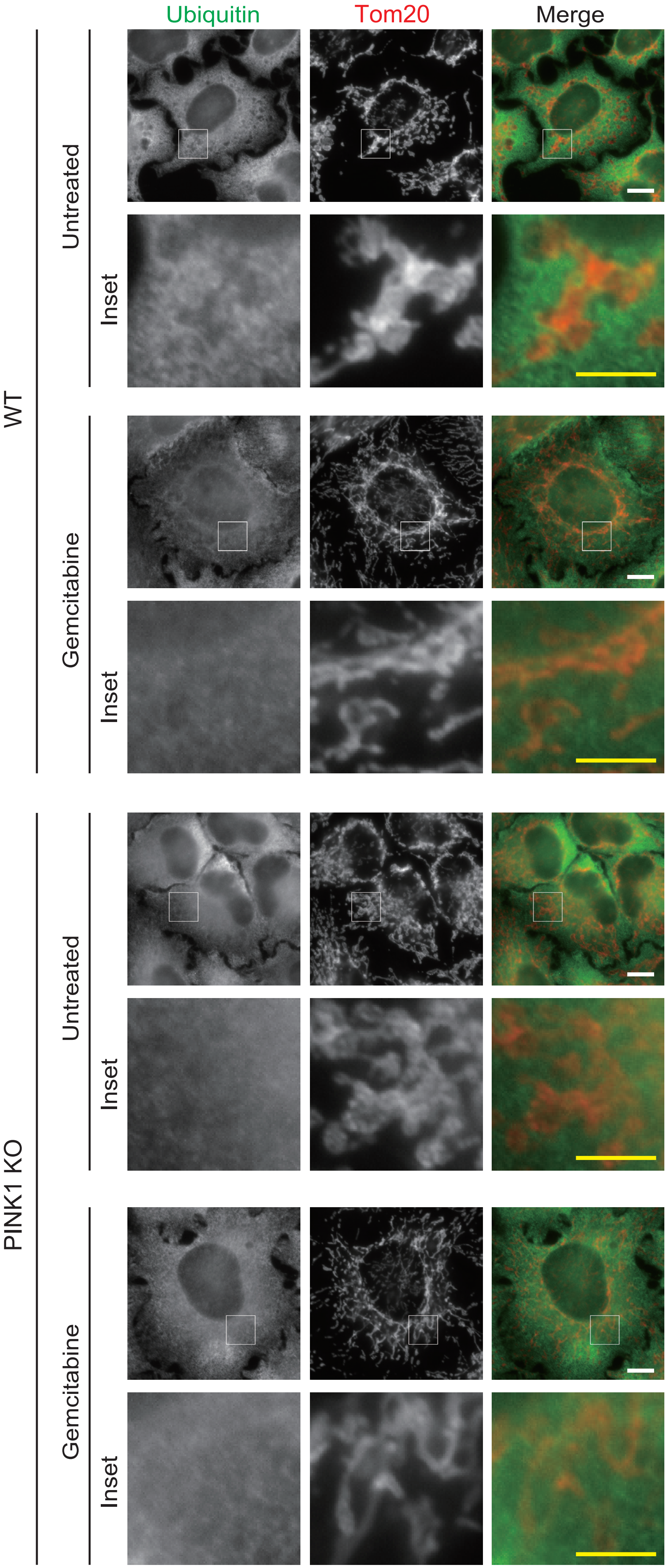

Figure S1 (continued)

G

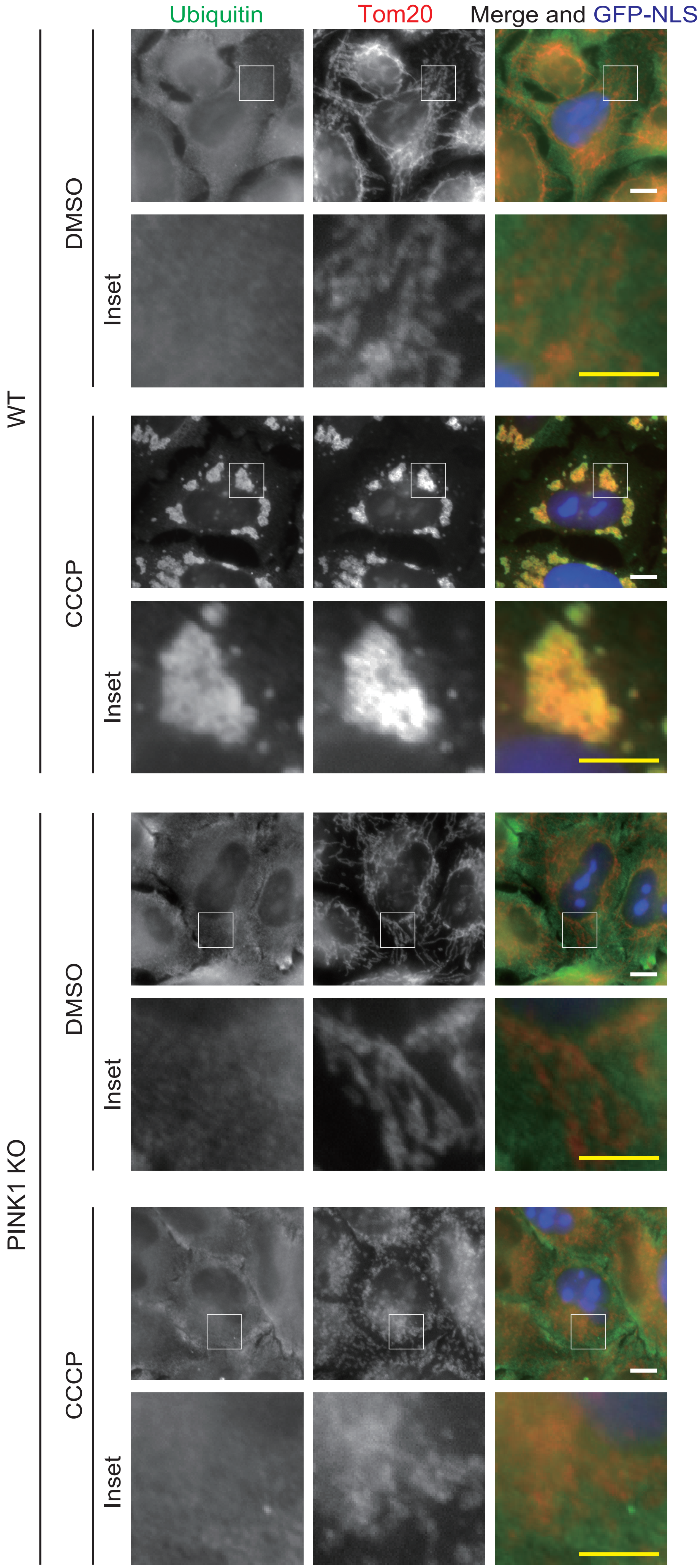

Figure S1 (continued)

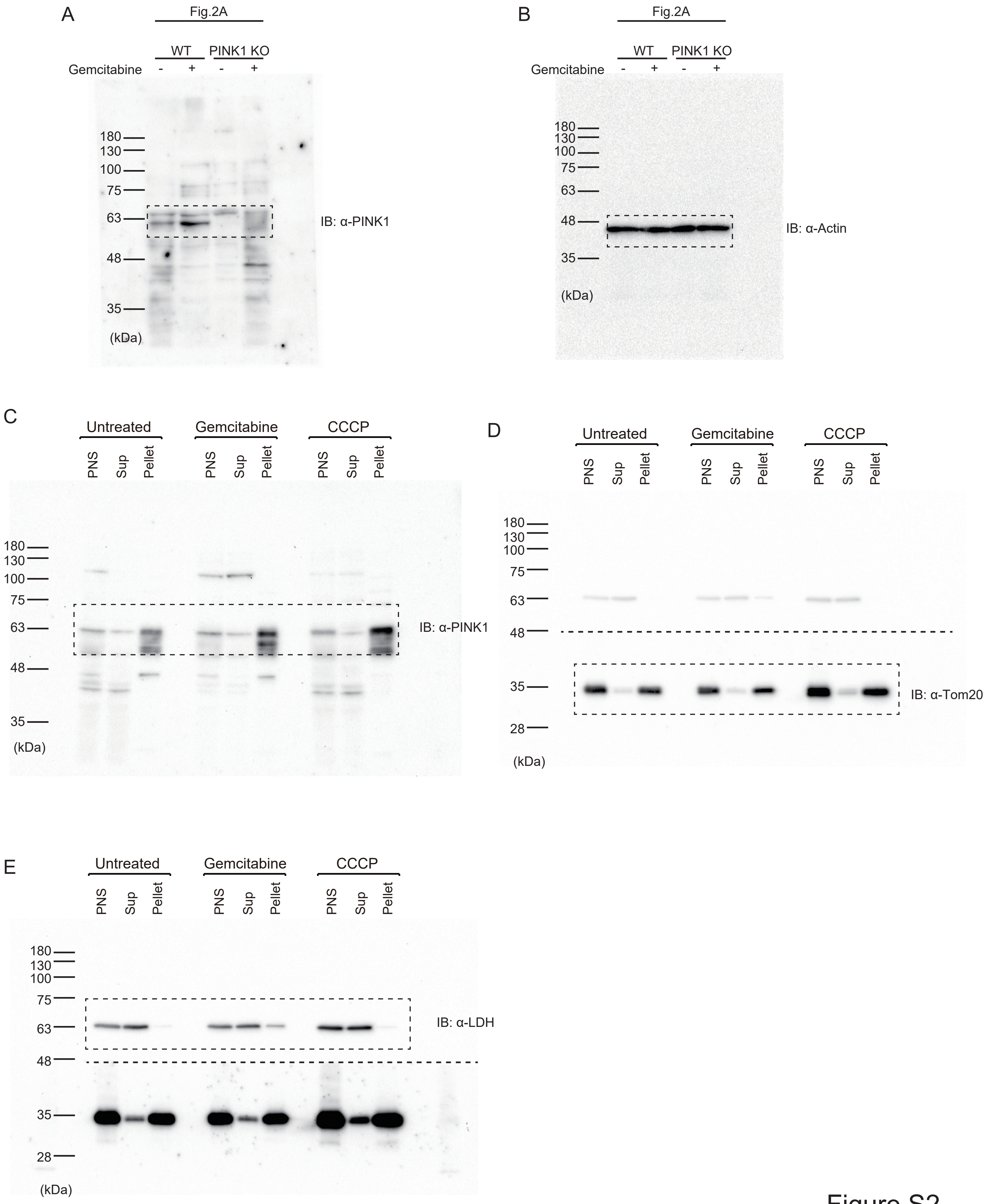
A
Fig.2A
WT
PINK1 KO
Gemcitabine - + - +
180
130
100
75
63
48
35
(kDa)
IB: α-PINK1
B
Fig.2A
WT
PINK1 KO
Gemcitabine - + - +
180
130
100
75
63
48
35
(kDa)
IB: α-Actin
C
Untreated
Gemcitabine
CCCP
PNS Sup Pellet PNS Sup Pellet PNS Sup Pellet
180
130
100
75
63
48
35
(kDa)
IB: α-PINK1
D
Untreated
Gemcitabine
CCCP
PNS Sup Pellet PNS Sup Pellet PNS Sup Pellet
180
130
100
75
63
48
35
28
(kDa)
IB: α-Tom20
E
Untreated
Gemcitabine
CCCP
PNS Sup Pellet PNS Sup Pellet PNS Sup Pellet
180
130
100
75
63
48
35
28
(kDa)
IB: α-LDH

Figure S2

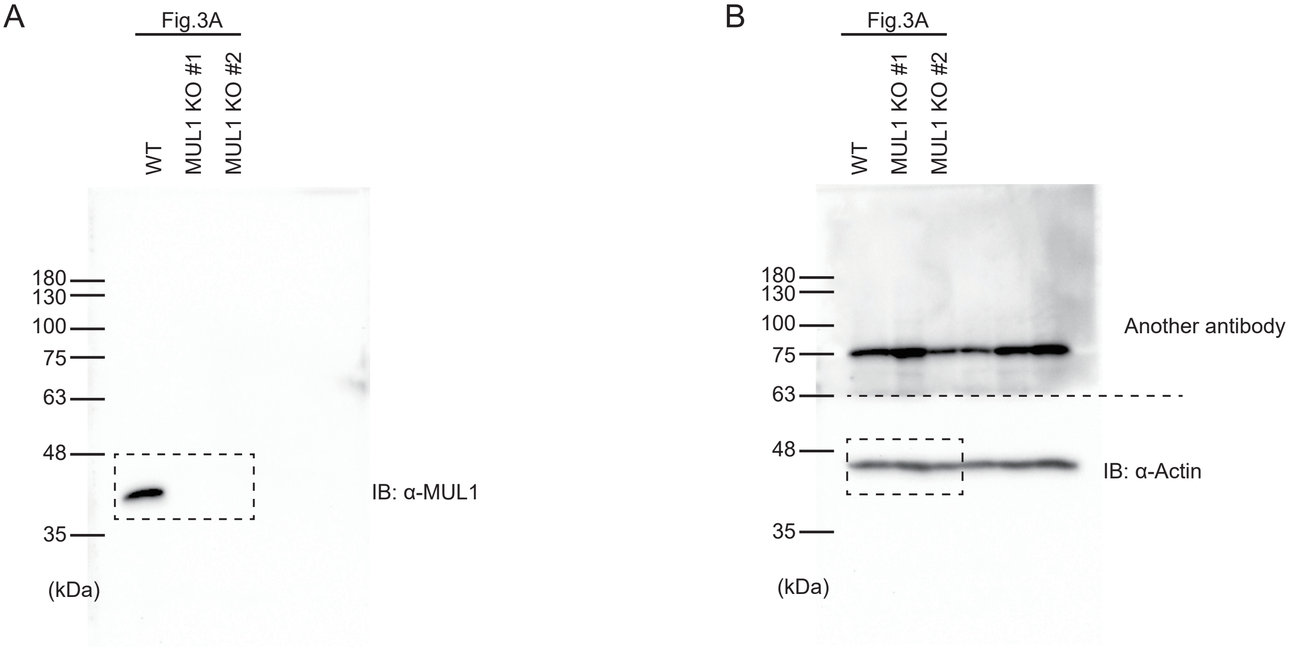
A
Fig.3A
WT
MUL1 KO #1
MUL1 KO #2
180
130
100
75
63
48
35
(kDa)
IB: α-MUL1
B
Fig.3A
WT
MUL1 KO #1
MUL1 KO #2
180
130
100
75
63
48
35
(kDa)
Another antibody
IB: α-Actin

Figure S3

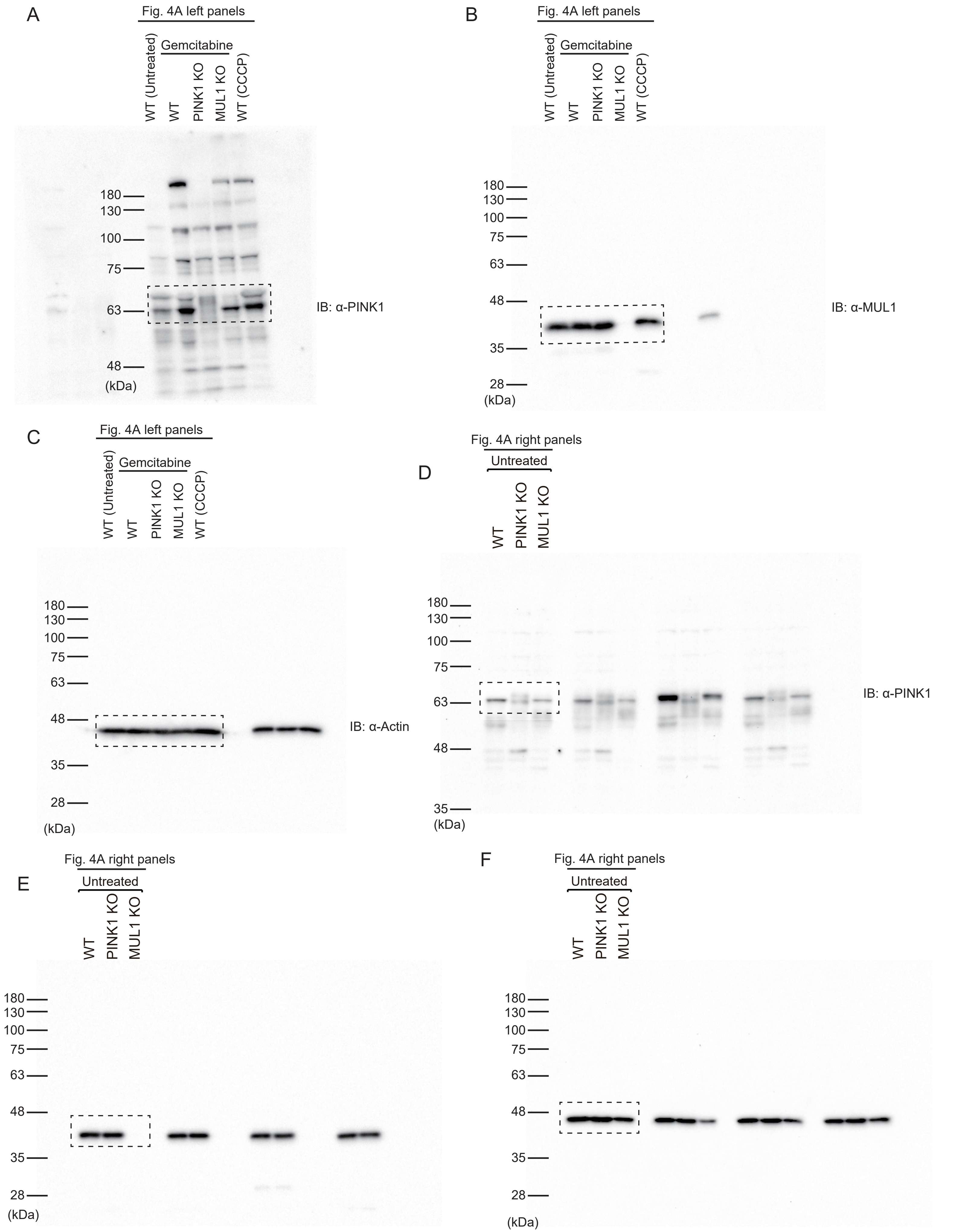

A
Fig. 4A left panels
WT (Untreated)
Gemcitabine
WT
PINK1 KO
MUL1 KO
WT (CCCP)
180
130
100
75
63
48
(kDa)
IB: α-PINK1
B
Fig. 4A left panels
WT (Untreated)
Gemcitabine
WT
PINK1 KO
MUL1 KO
WT (CCCP)
180
130
100
75
63
48
35
28
(kDa)
IB: α-MUL1
C
Fig. 4A left panels
WT (Untreated)
Gemcitabine
WT
PINK1 KO
MUL1 KO
WT (CCCP)
180
130
100
75
63
48
35
28
(kDa)
IB: α-Actin
D
Fig. 4A right panels
Untreated
WT
PINK1 KO
MUL1 KO
180
130
100
75
63
48
35
(kDa)
IB: α-PINK1
E
Fig. 4A right panels
Untreated
WT
PINK1 KO
MUL1 KO
180
130
100
75
63
48
35
28
(kDa)
IB: α-MUL1
F
Fig. 4A right panels
Untreated
WT
PINK1 KO
MUL1 KO
180
130
100
75
63
48
35
28
(kDa)
IB: α-Actin

Figure S4

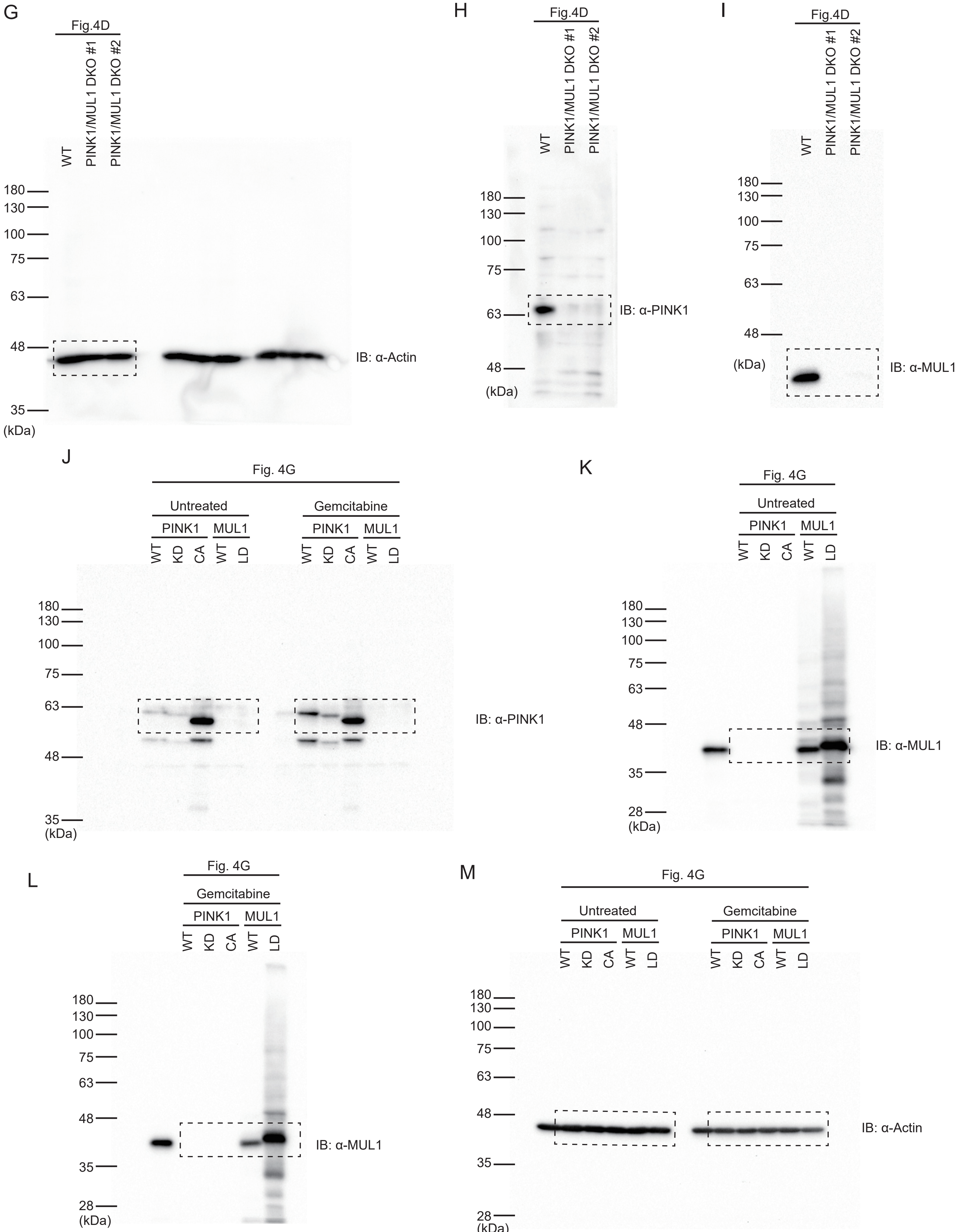

Figure S4 (continued)
