# Supplementary material for: Gemcitabine induces Parkin-independent mitophagy through mitochondrial-resident E3 ligase MUL1-mediated stabilization of PINK1
Source: Sci Rep. 2020 Jan 30;10:1465. doi: 10.1038/s41598-020-58315-w (PMC6992789; doi:10.1038/s41598-020-58315-w)
Supplement: Supplementary file 2 — Supplementary Information 2. [file 41598_2020_58315_MOESM2_ESM.docx]

**Supplementary information**

**Gemcitabine induces Parkin-independent mitophagy through mitochondrial-resident E3 ligase MUL1-mediated stabilization of PINK1**

Ryoko Igarashi^13^, Shun-ichi Yamashita^1*^, Tomohiro Yamashita^2^, Keiichi Inoue^1^, Tomoyuki Fukuda^1^, Takeo Fukuchi^3^, Tomotake Kanki^1*^

^1^Department of Cellular Physiology, Niigata University Graduate School of Medical and Dental Sciences, Niigata 951-8510

^2^Department of Global Healthcare, Graduate School of Pharmaceutical Sciences, Kyushu University, Fukuoka, Japan

^3^Department of Ophthalmology, Niigata University Graduate School of Medical and Dental Sciences, Niigata 951-8510, Japan

Figure S1

Full-length blots of PINK1 (A), ATG14 (C), and Actin (B, D) for Fig. 1. The areas enclosed by the dotted rectangles are cropped and shown in Fig. 1C and E. (E) Cells were cultured with 100 μM gemcitabine for 48 h. To accumulate autophagosomes, cells were treated with 100 nM bafilomycin A1 for the last 12 h of gemcitabine treatment. After treatment, cells were fixed and stained with anti-LC3 and anti-Tom20 antibodies. Colocalization of Tom20 and LC3 is indicated by yellow arrows. (F, G) Cells were cultured with 100 μM gemcitabine for 48 h (F) or with 10 μM CCCP for 3 h (G), then fixed and stained with anti-ubiquitin and anti-Tom20 antibodies. For CCCP treatment, cells were transfected with Parkin-IRES-GFP-NLS vector. Bars: white bars, 10 μm; yellow bars, 5 μm.

Figure S2

Full-length blots of PINK1 (A) and Actin (B) for Fig. 2A. The areas enclosed by the dotted rectangles are cropped and shown in Fig. 2A. Full-length blots of PINK1 (C), Tom20 (D), and LDH (E) for Fig. 2B. The areas enclosed by the dotted rectangles are cropped and shown in Fig. 2B.

Figure S3

Full-length blots of MUL1 (A) and Actin (B) for Fig. 3. The areas enclosed by the dotted rectangles are cropped and shown in Fig. 3A.

Figure S4

Full-length blots of PINK1 (A, D, H, J), MUL1 (B, E, I, K, L), and Actin (C, F, G, M) for Fig. 4. The areas enclosed by the dotted rectangles are cropped and shown in Fig. 4A, C and F.
